# Biomechanical analysis of subcondylar fracture fixation using miniplates at different positions and of different lengths

**DOI:** 10.1186/s12903-021-01905-5

**Published:** 2021-10-21

**Authors:** Chao-Min Huang, Man-Yee Chan, Jui-Ting Hsu, Kuo-Chih Su

**Affiliations:** 1 Department of Stomatology, Taichung Veterans General Hospital, Taichung, 407 Taiwan; 2 Department of Dentistry, Taichung Tzu Chi Hospital, Buddhist Tzu Chi Medical Foundation, Taichung, 427 Taiwan; 3 School of Dentistry, College of Oral Medicine, Chung Shan Medical University, Taichung, 402 Taiwan; 4 School of Dentistry, China Medical University, Taichung, 404 Taiwan; 5 Department of Medical Research, Taichung Veterans General Hospital, Taichung, 407 Taiwan; 6 Department of Biomedical Engineering, Hungkuang University, Taichung, 433 Taiwan; 7 Department of Chemical and Materials Engineering, Tunghai University, Taichung, 407 Taiwan

**Keywords:** Subcondylar fracture, Open reduction and internal fixation, Miniplates

## Abstract

**Background:**

Many types of titanium plates were used to treat subcondylar fracture clinically. However, the efficacy of fixation in different implant positions and lengths of the bone plate has not been thoroughly investigated. Therefore, the primary purpose of this study was to use finite element analysis (FEA) to analyze the biomechanical effects of subcondylar fracture fixation with miniplates at different positions and lengths so that clinicians were able to find a better strategy of fixation to improve the efficacy and outcome of treatment.

**Methods:**

The CAD software was used to combine the mandible, miniplate, and screw to create seven different FEA computer models. These models with subcondylar fracture were fixed with miniplates at different positions and of different lengths. The right unilateral molar clench occlusal mode was applied. The observational indicators were the reaction force at the temporomandibular joint, von Mises stress of the mandibular bone, miniplate and screw, and the sliding distance on the oblique surface of the fracture site at the mandibular condyle.

**Results:**

The results showed the efficacy of fixation was better when two miniplates were used comparing to only one miniplates. Moreover, using longer miniplates for fixation had better results than the short one. Furthermore, fixing miniplates at the posterior portion of subcondylar region would have a better fixation efficacy and less sliding distance (5.46–5.76 μm) than fixing at the anterolateral surface of subcondylar region (6.10–7.00 μm).

**Conclusion:**

Miniplate fixation, which was placed closer to the posterior margin, could effectively reduce the amount of sliding distance in the fracture site, thereby achieving greater stability. Furthermore, fixation efficiency was improved when an additional miniplate was placed at the anterior margin. Our study suggested that the placement of miniplates at the posterior surface and the additional plate could effectively improve stability.

## Background

Mandibular fractures are a common form of facial bone fractures caused by trauma to the facial area in traffic accidents, falls, and physical violence [1]. Condylar fractures account for approximately 18–42% of total cases of mandibular fracture, and subcondylar fractures account for approximately half of all condylar fractures [1–3]. If a subcondylar fracture involves displacement, changes in the occlusal relationship can occur; surgery is usually recommended to reduce the fracture. The treatment of subcondylar fractures includes both closed and open reduction, as well as internal fixation. Closed reduction involves using intermaxillary fixation to reduce the displacement of the mandibular body and fragment of condylar fracture, to achieve better osseointegration. In open reduction and internal fixation, an endoscope is used to enter the subcondylar fracture region via retromolar approach intraorally to reduce the fracture. Another way to perform open reduction and internal fixation is via extraoral approach to reduce them. Moreover, a titanium plate is used for the internal fixation of the fracture site, thereby achieving therapeutic goals and achieving osteointegration [4]. Patients, who underwent open reduction and internal fixation, exhibited better surgical outcomes, including larger maximum mouth opening and better intermaxillary relationship [5–7].

An I-shaped titanium bone plate is often clinically used for fixation because of its high flexibility and malleability, which helps achieve passive adaptation to the fracture surface [8]. However, bone plates used in clinics differ owing to different implantation conditions; therefore, clinicians may select plates of different lengths or quantities. Although bone plate fixation can help patients achieve better osteointegration and prognosis, currently, no literature exists on the differences in the number, position, and length of bone plate implantation or their biomechanical evaluation.

Several researchers have previously aimed to evaluate the effect of bone plate implantation and biomechanical analysis, to develop more effective strategies for bone plate fixation [9–12]. A clinical study by Marwan et al. [9] showed that using two bone plates for subcondylar fracture fixation resulted in a better prognosis and fewer complications than using a single bone plate. Cimen et al. [10] used biomechanical evaluation to analyze the post-implantation effects of single-titanium and double-titanium miniplates, and found that double-titanium miniplate implantation resulted in better stability. Using finite element analysis (FEA) has proven appropriate for investigating the effects of miniplate implantation in the mandible. Hijazi et al. [13] evaluated the effects of various occlusal conditions, such as incisal clench, intercuspal position, molar clench, and group function during double titanium miniplate implantation via FEA. Notably, higher contralateral occlusal stress was induced during a contralateral occlusion task, with higher stress on the fracture and bone plate on the ipsilateral side. Aquilina et al. [14] also used FEA to evaluate the differences between different types of bone plates (straight, rectangular, square, and X plates) after implantation, and evaluated post-implantation stability using the degree of bone displacement.

Based on the literature, the effects of different numbers of bone plates have been evaluated, but the different implant positions and lengths of the bone plate have not. Therefore, the primary purpose of this study was to use FEA to investigate the biomechanical effects of subcondylar fracture fixation with miniplates at different positions and lengths. The findings will provide clinicians with a biomechanical basis for treating subcondylar fractures and selecting bone plates, which, in turn, could improve the therapeutic success rate and prognosis.

## Methods

### Build a simulation geometry model

**Fig. 1:**
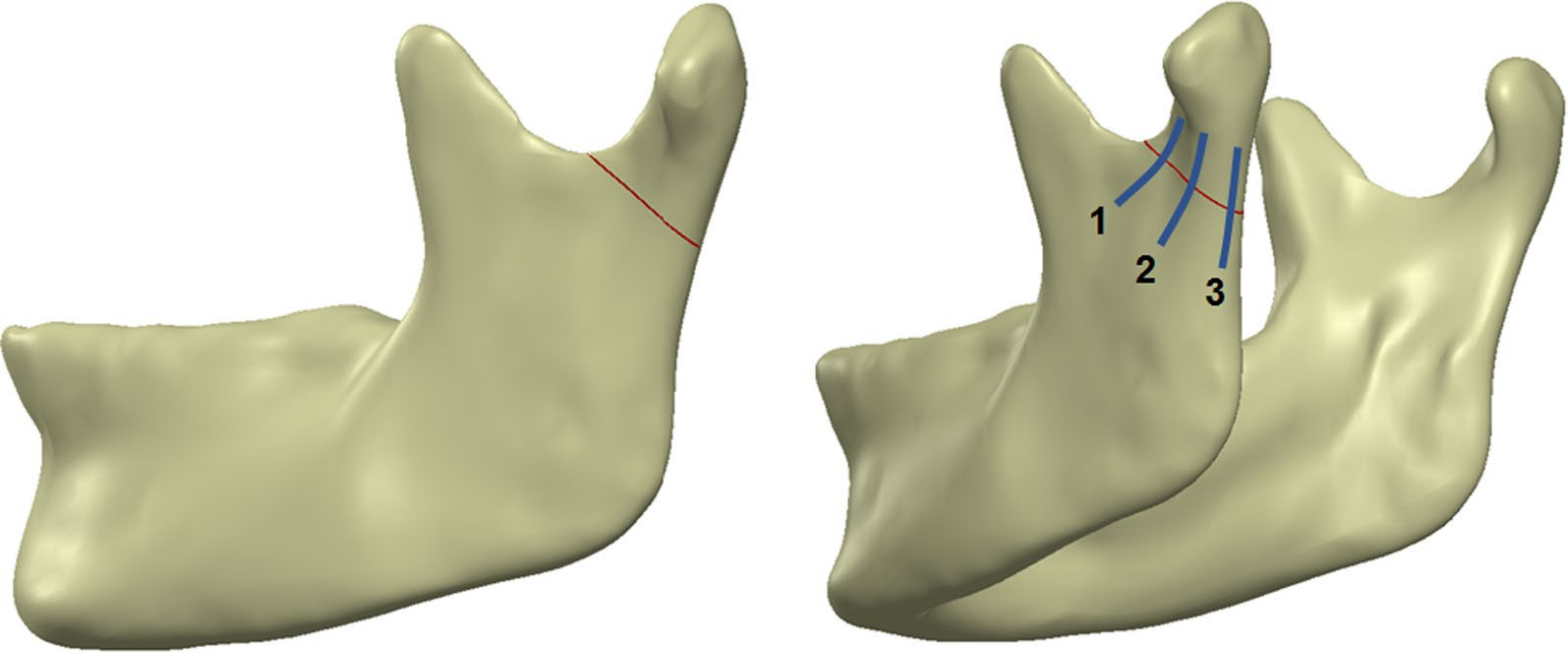
Subcondylar fracture and different miniplate implantation positions (1. anterolateral surface. 2. posterolateral surface of the mandibular condyle. 3. posterior surface of the mandibular condyle)

In this study, an FEA computer model was developed to investigate the biomechanics of subcondylar fracture fixation using miniplates at different positions and lengths. The computer model used here included four major structures: mandibular cortical bone, mandibular trabecular bone, miniplate and screw. The model of the mandible was established using CT images obtained from the Visible Human Project of the United States National Institutes of Health. Medical image reconstruction software (Mimics Medical 20.0, Materialise, Leuven, Belgium) was used to reconstruct the segmentation of the human mandible using CT images. Medical Image Reconstruction Software was used to proceed with the CT image by setting the threshold of the grayscale values between 226 to 3071 HU to obtain the outline of the mandible bone. During the procedure, some artifacts were also present. Next, the artifacts were removed and the regions of the trabecular bone were designated manually slice by slice. 3D computer-aided design (CAD) software (Solidworks 2016, Dassault Systemes SolidWorks Corp, Waltham, MA, USA) was used to construct the model of the miniplate; there were two types of miniplates: a long miniplate (four holes) and short miniplate (two holes). Moreover, CAD software was used to create an oblique fracture site at the mandibular condyle and reduction had been performed (Fig. 1).

**Fig. 2:**
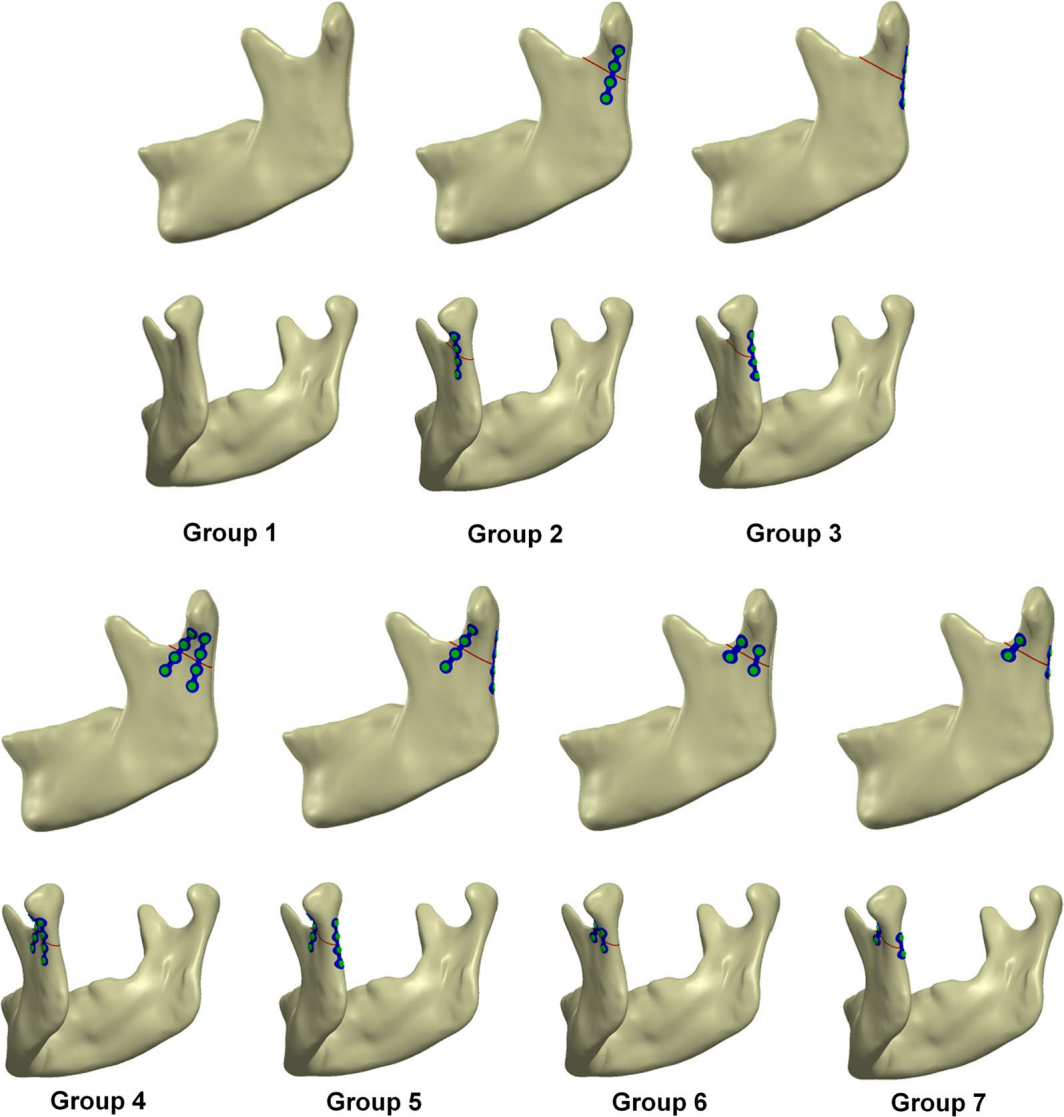
Seven different FEA models for subcondylar fracture fixation using miniplates at different positions and of different lengths. Group 1 was the control group. Group 2–7 were the experimental group and internal fixation was performed by different fixation strategies

In this study, different miniplate implant positions—at the anterolateral surface of the mandibular condyle (Fig. 1 position 1), posterolateral surface of the subcondylar region (Fig. 1 position 2), and posterior surface of the subcondylar region (Fig. 1 position 3)—and two different miniplate lengths were used. The fracture line was placed at the lowest midportion of the sigmoid notch and extended to the posterior border of the mandible. The CAD software combined the mandible, miniplate, and screws to create seven different FEA computer models (Fig. 2) of an intact mandible structure as Group 1. Group 2 contained a left subcondylar fracture with internal fixation at the posterolateral surface of the subcondylar region using a long miniplate; meanwhile, Group 3 included a left subcondylar fracture with internal fixation at the posterior surface of the subcondylar region using a long miniplate. In Group 4, the internal fixation was at the anterolateral and posterolateral surface of the subcondylar region using a long miniplate. In Group 5, the internal fixation was at the anterolateral and the posterior surface of the subcondylar region using a long miniplate. In Group 6, the internal fixation was at the anterolateral and posterolateral surface of the subcondylar region using a short miniplate. Finally, Group 7 had a left subcondylar fracture with internal fixation at the anterolateral and the posterior surface subcondylar region using a short miniplate. The seven groups were imported into FEA software (ANSYS Workbench 18.0, ANSYS, Inc., Canonsburg, PA) for the analysis.

### Loading conditions and boundary conditions

**Fig. 3:**
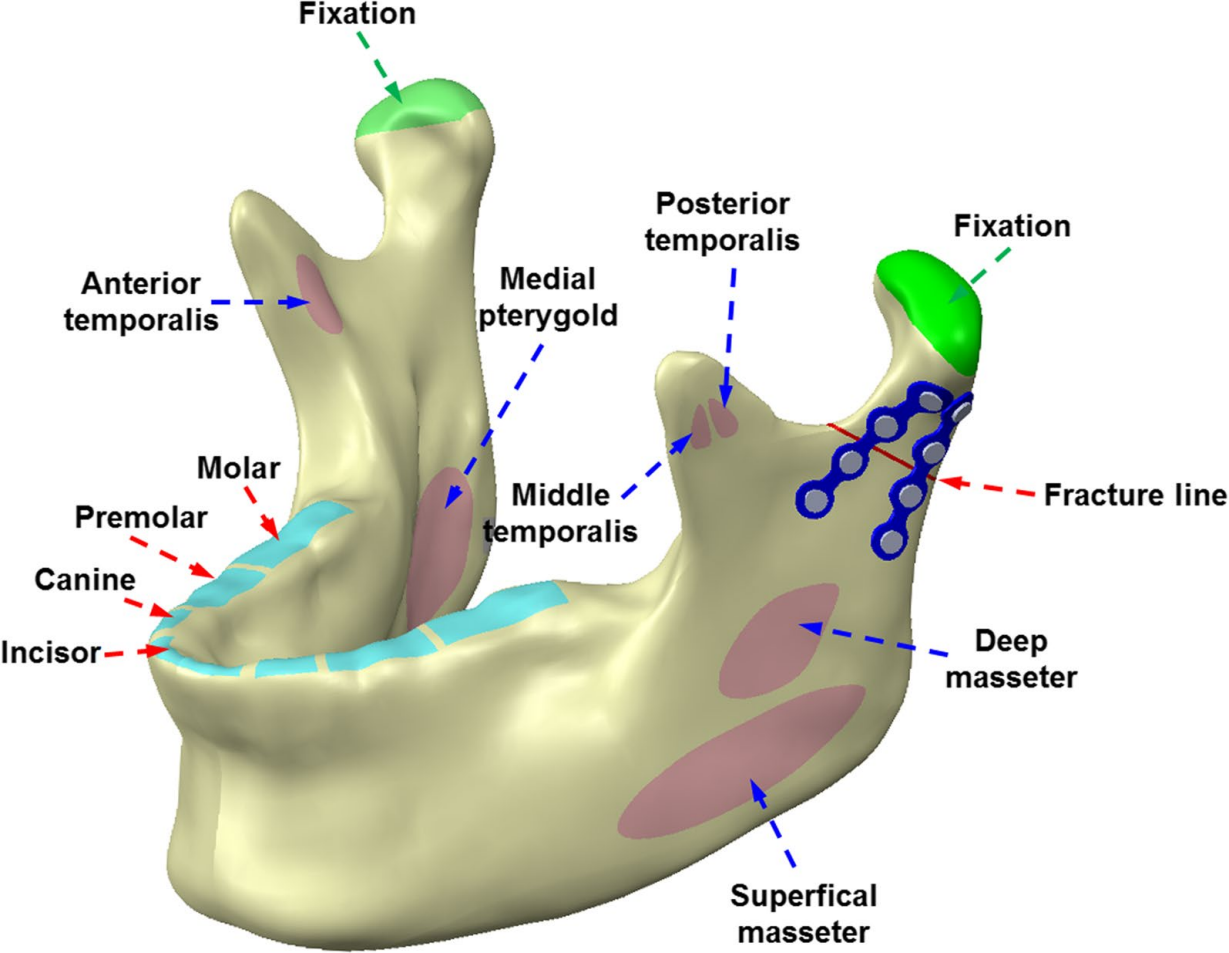
Position of loading conditions and boundary conditions in finite element analysis model

**Fig. 4:**
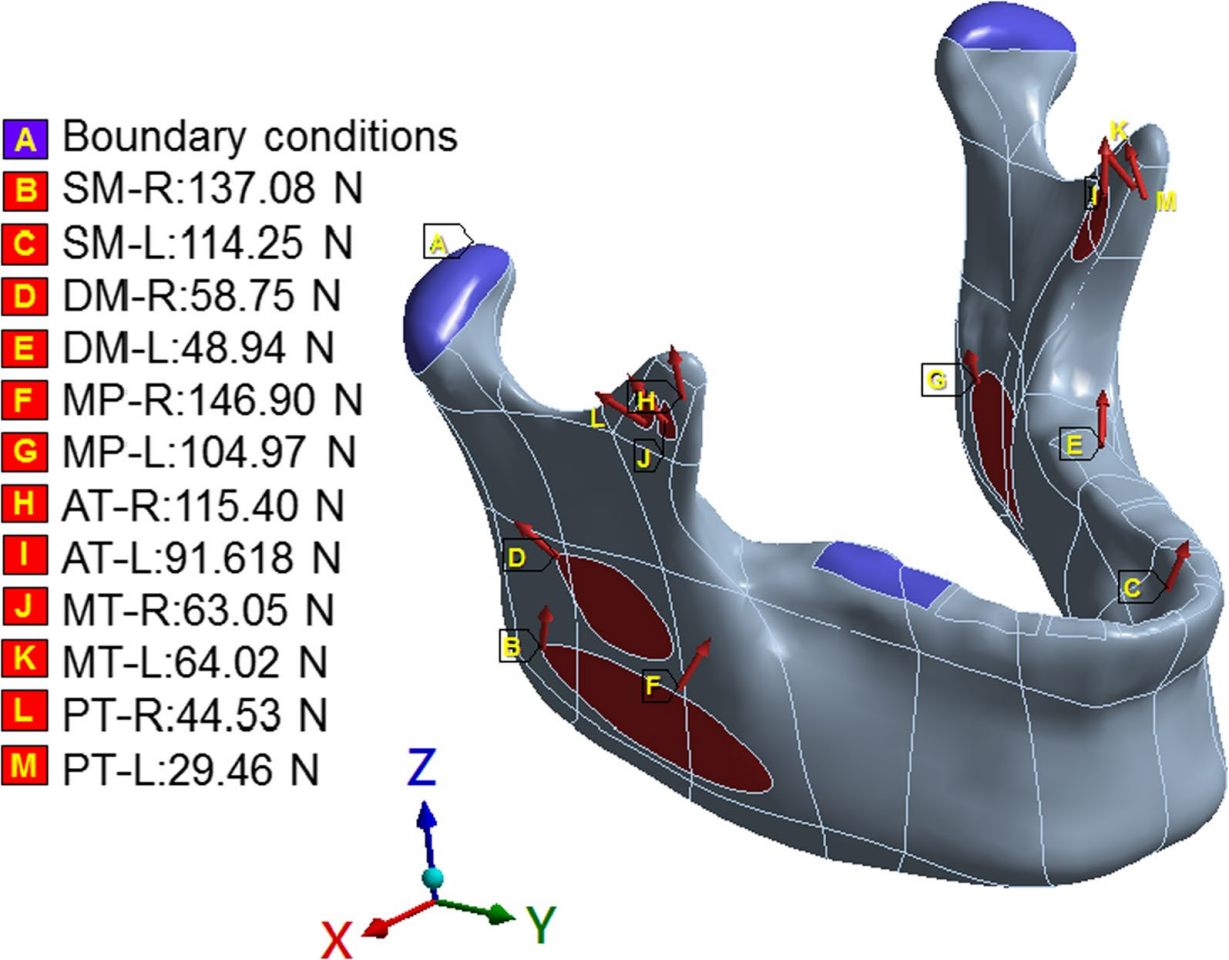
Effect of muscle force during right unilateral molar clench

This study was based primarily on previous studies. The contralateral occlusion task has relatively high contralateral occlusal stress [11]; therefore, herein, the right unilateral molar clench (RMOL) occlusal mode was used. The loading conditions were set to the external forces exerted by the superficial masseter (SM), deep masseter (DM), medial pterygoid (MP), anterior temporalis (AT), middle temporalis (MT), and posterior temporalis (PT) (Fig. 3); the magnitude and direction of these external forces are shown in Table 1 [15, 16]. The boundary conditions were set with the temporomandibular condyle as a fixed end, and the X-, Y-, and Z-axis displacements at this site were set as 0. The position of the right molar was fixed to simulate the condition of the tooth during RMOL, which simulated contact with the right (unilateral) posterior tooth (Fig. 4). Additionally, for contact between the miniplate and screw, the contact between the miniplate and mandible was set to “no separation,” which simulated the surface when there is no separation and only a small amount of frictionless sliding was allowed [17]. The contact at the mandibular condyle oblique fracture site was set to frictional; the friction coefficient was set to 0.45 [18].

**Table 1:** Simulation of loading conditions on the teeth during RMOL

Side	Direction	Muscular force (N)						Constrained area
		SM	DM	MP	AT	MT	PT	
Right	Force	137.1	58.8	146.8	115.3	63.1	44.6	Constrained the right molars
	Fx	28.4	32.1	− 71.4	17.2	14.0	9.3	
	Fy	57.4	− 21.0	54.8	5.1	− 31.5	− 38.1	
	Fz	121.2	44.5	116.1	114.0	52.8	21.1	
Left	Force	114.2	49.0	104.9	91.6	64.1	29.5	
	Fx	− 23.6	− 26.7	51.0	− 13.7	− 14.2	− 6.1	
	Fy	47.9	− 17.5	39.1	4.0	− 32.0	− 25.2	
	Fz	101.0	37.1	83.0	90.5	53.6	14.0	

Magnitude and direction of the external forces from each muscle under different occlusal modes [15, 16]

### Material properties of the model

**Fig. 5:**
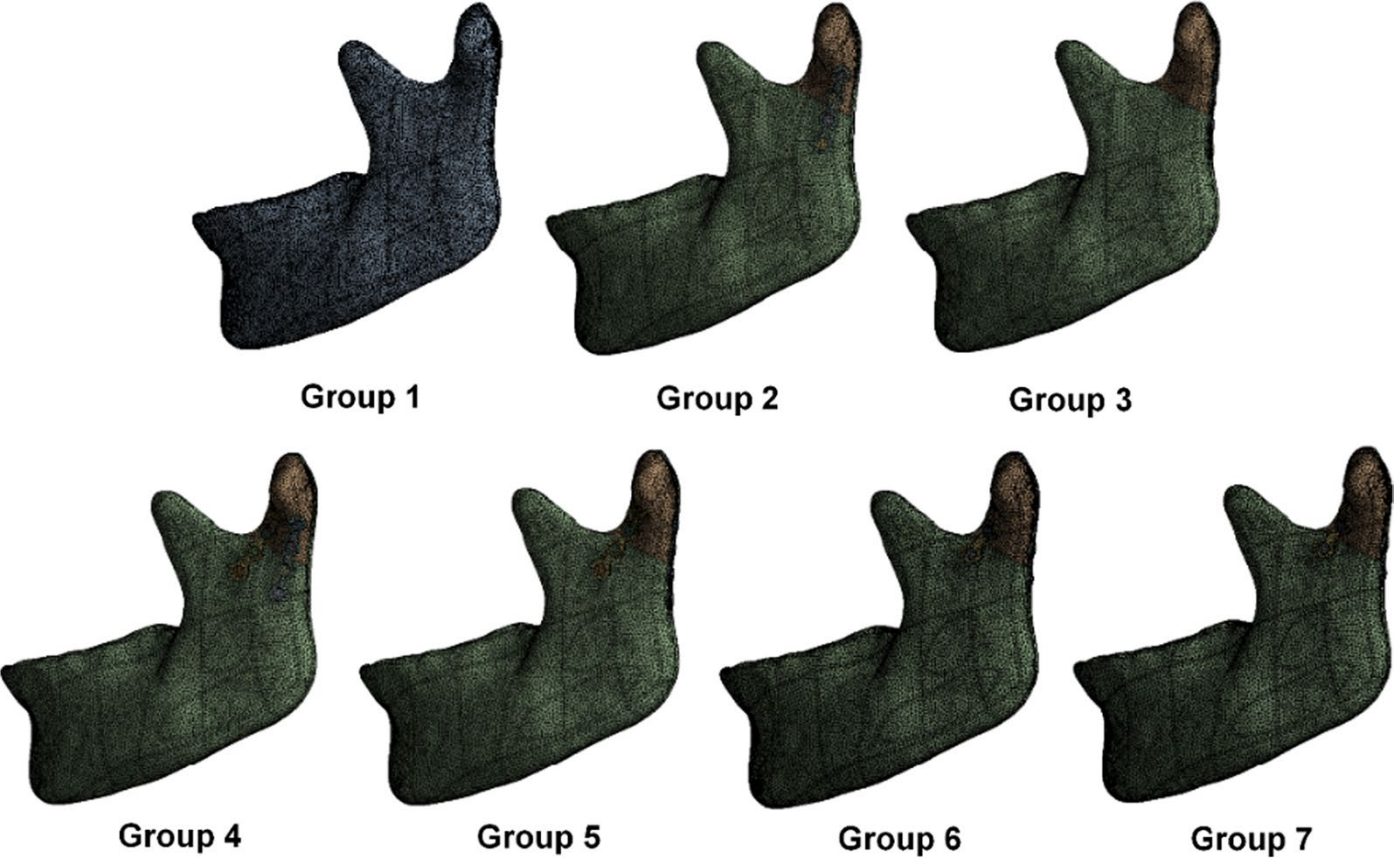
Computer model mesh for this study

The model comprised four parts: cortical bone, trabecular bone, miniplate, and screws. The materials properties used in this simulation, which are listed in Table 2, were sourced from available literature [19]. All materials were assumed to be homogeneous, isotropic, and linearly elastic; consequently, two independent parameters—Young’s modulus (E) and Poisson’s ratio (ν)—were used to express the material properties. The material of the screws in this study was titanium alloy, and the material of miniplates was pure titanium, which both were the same as in clinical (Table 2). Additionally, the FEA computer model used a 0.5 mm tetrahedral mesh, as shown in Fig. 5. After conducting the convergence test on the meshes, all models reached the 5% stop criteria for this test [20]. Table 3 shows the number of nodes and elements in each group.

**Table 2:** Material property settings for this study

		Young's modulus (MPa)	Poisson's ratio
Trabecular bone		1000	0.3
Cortical bone		17,000	0.3
Miniplate	Pure titanium	110,000	0.3
Screw	Titanium alloy	118,000	0.3

**Table 3:** Number of nodes and elements for each group

Mesh number	Group 1	Group 2	Group 3	Group 4	Group 5	Group 6	Group 7
Nodes	684,452	709,591	719,179	729,760	740,522	706,493	707,131
Elements	396,042	410,148	415,786	420,778	427,214	407,439	407,898

For FEA, the observational indicators were the reaction force at the temporomandibular joint (TMJ) and von Mises stress of the mandibular bone, miniplate, and screw, and the maximum sliding distance on the oblique surface of the fracture site at the mandibular condyle. The sliding distance is defined as the displacement between the surfaces of two fracture fragments. That is, the higher the sliding distance, the higher the delamination of debonding. This study used the built-in calculation tools provided by ANSYS Workbench to solve the sliding distance between two surfaces of the condylar fracture fragments.

## Results

The distribution of the von Mises stress in the overall structure, reaction force at the left and right fixed ends of the TMJ, and sliding distance on the oblique surface of the fracture site at the mandibular condyle were obtained using the FEA.

**Fig. 6:**
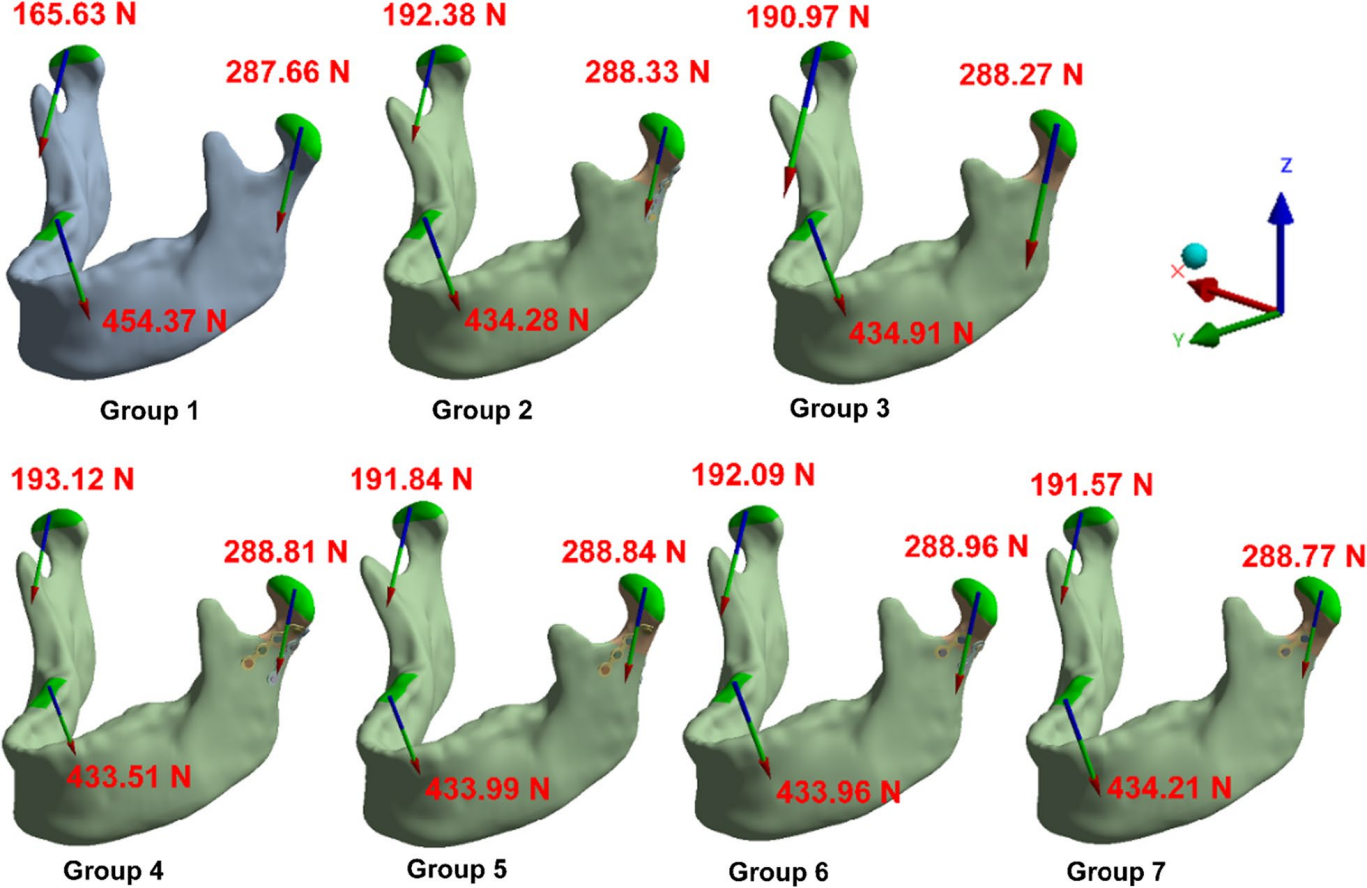
Illustrations of magnitude and direction of the reaction force on the left and right TMJ in each group

Figure 6 shows the magnitude and direction of the reaction force on the TMJ after the subcondylar fracture fixation. The contralateral TMJ on the occlusal side was affected by a greater external force. The occlusal side was also affected by the miniplate fixation on the contralateral side with a higher reaction force than the intact mandibular structure. The reaction force on the right side of the TMJ in groups 2–7 all exceeded 190.00 N compared to group 1, which was only 165.60 N.

Table 4 shows the component forces of the reaction force on the left and right TMJ in each group and the force exerted along each axis. The reaction force was higher on the left side than the right, 287.66 N comparing to 165.63 N in group 1. The trend remained in all experimental groups in which the internal fixation was applied on the condylar fracture. Furthermore, among all the experimental groups, the increasing amount of the reaction force compared to the control group at the right side was much greater than the left side (e.g., the increasing amount on the right side comparing group 2 to group 1 was 26.67 N, and 0.67 N on the left side.).

**Table 4:** Magnitude and direction of the reaction force on the left and right TMJ in each group

		Group 1	Group 2	Group 3	Group 4	Group 5	Group 6	Group 7
Right	X	− 20.19 N	− 25.07 N	− 24.84 N	− 25.15 N	− 24.94 N	− 24.95 N	− 24.91 N
	Y	77.75 N	87.00 N	86.53 N	87.28 N	86.80 N	86.95 N	86.75 N
	Z	− 144.84 N	− 169.74 N	− 168.41 N	− 170.42 N	− 169.23 N	− 169.45 N	− 168.97 N
	Total	165.63 N	192.3 N	190.97 N	193.12 N	191.84 N	192.09 N	191.57 N
Left	X	36.14 N	35.98 N	34.76 N	36.68 N	35.62 N	36.03 N	35.39 N
	Y	46.14 N	46.25 N	45.70 N	46.40 N	45.92 N	46.26 N	45.89 N
	Z	− 281.62 N	− 282.32 N	− 282.50 N	− 282.68 N	− 282.93 N	− 282.94 N	− 282.89 N
	Total	287.66 N	288.33 N	288.27 N	288.81 N	288.84 N	288.96 N	288.77 N

**Fig. 7:**
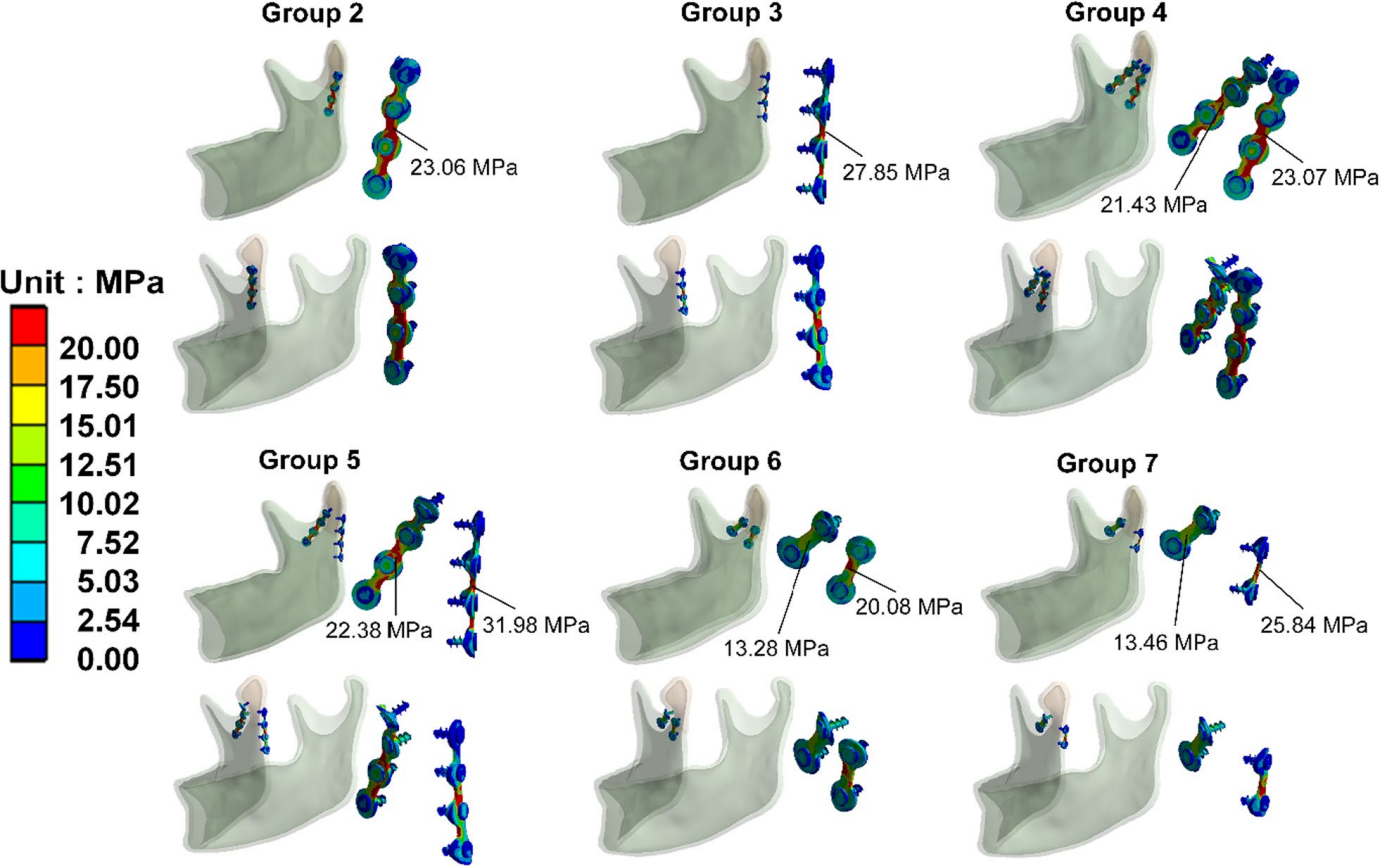
Von Mises stress distribution on the miniplates and screws in each group

Figure 7 shows the von Mises stress distribution on the miniplates and screws in each group. The miniplates were shown to be near the oblique surface of the fracture site have higher stress. Additionally, when the two-miniplate groups (Groups 4, 5, 6, and 7) were tested, the miniplate closer to the posterior side experienced a higher stress distribution. (23.07 MPa compared to 21.43 MPa, 31.98 MPa compared to 22.38 MPa, 20.08 MPa compared to 13.28 MPa, 25.84Mpa compared to 13.46 MPa, respectively).

**Fig. 8:**
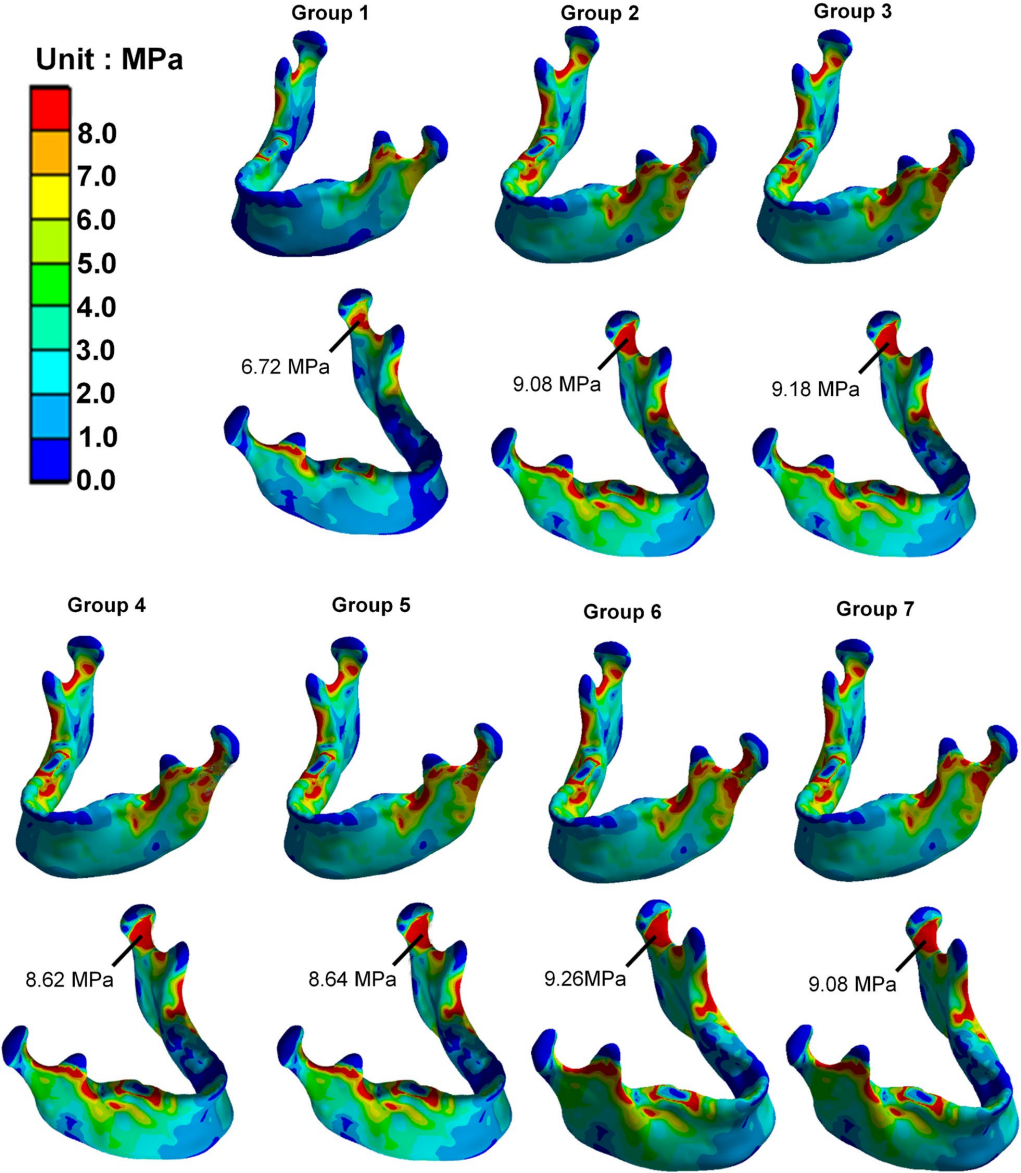
Von Mises stress distribution on the overall structure of the mandibular bone in each group

Figure 8 shows the von Mises stress distribution in each group after the subcondylar fracture fixation. In the intact mandibular structure (Group 1), the RMOL occlusal mode caused relatively high stress on the occlusal mandibular condyle. Furthermore, when the other groups were tested, there was relatively high stress on the mandibular condyle contralateral to the occlusion when a miniplate was used for the subcondylar fracture fixation; however, the differences in the stress distribution of each group were minor.

**Fig. 9:**
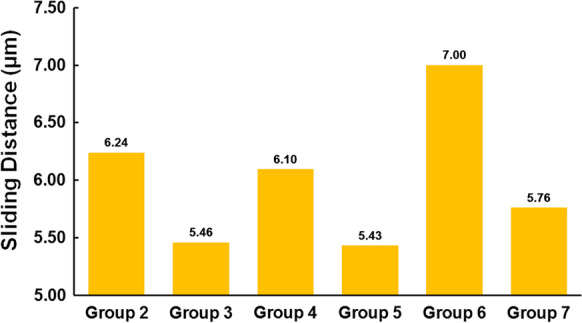
Maximum sliding distance of the oblique surface on the mandibular condyle fracture site

Figure 9 shows the maximum sliding distance on the oblique surface of the fracture site on the mandibular condyle after subcondylar fracture fixation using a miniplate. The figure shows a larger sliding distance on the oblique surface of the fracture site in Groups 2, 4, and especially, Group 6 (6.24 µm, 6.09 µm, 7.00 µm, respectively). The sliding distance on the oblique surface of the fracture site in Groups 3, 5, and 7 was relatively small (5.46 µm, 5.43 µm, 5.76 µm, respectively).

## Discussion

Using miniplate fixation for subcondylar fractures has become a common clinical treatment, and the outcomes were well acceptable [21]. Although some physicians and researchers have conducted studies on the efficacy of miniplate fixation, most existing mechanical studies have not considered the influence of fixation using miniplates at different positions and of different lengths. Furthermore, the mechanical analysis was more complicated for such fixation. Thus, in this study, FEA was successfully used for the biomechanical analysis of fixation using miniplates at different positions and lengths. Consequently, the results can enable physicians and researchers to understand the effects of such fixation.

In the FEA, the reaction force at the fixed end of the TMJ was observed owing to the selected boundary conditions. When the mandible is not affected by the subcondylar fracture, a relatively high reaction force is produced on the contralateral TMJ when the mandible is subjected to RMOL. Therefore, this study investigated the effects of implanting different miniplates for contralateral subcondylar fracture fixation. Notably, in unilateral subcondylar fracture fixation using a miniplate, the reaction force of the subcondylar fracture (the side fixed with the miniplate) did not increase considerably during the contralateral molar clench. Conversely, the reaction force on the TMJ on the occlusal side was increased from 165.63 N to 193.12 N. Among groups 2 to 7, the increased amount of the force to the medial, anterior and inferior direction along with the X-, Y-, Z-axis respectively were observed on the right side (e.g.,4.88 N, 9.25 N, 23.42 N of the force toward the medial, anterior, and inferior direction respectively increased in group 2 compared to group 1.). On the other hand, the reaction force on the left changed very little. (e.g., increased 0.67 N of reaction force in group 2 compared to group 1.) (Table 4) It suggested that the alteration of stress distribution would be more remarkable on the unaffected side of the mandible.

Herein, RMOL occlusion in contralateral subcondylar fracture was selected to evaluate the effects of fixation using miniplates at different positions and of different lengths. There was little difference in the stress distribution on the mandible in subcondylar fracture fixation. However, there was higher stress on the mandibular condyle on the side opposite of the occlusion and the result was consistent with the conclusion made by Hijazi et al. which showed higher contralateral occlusal stress was induced during a contralateral occlusion task, with higher stress on the fracture and bone plate on the ipsilateral side [13]. The von Mises stress distribution of the mandibular body was observed. In the absence of mandibular bone fracture, the stress was mainly concentrated near the coronoid process. After the subcondylar fracture and miniplate fixation, the changes in stress distribution were observed to be concentrated in the subcondylar fracture area and anterior condyle head; the overall distribution was more uneven than with no fracture. Additionally, the stress distribution of the mandibular body did not differ much between different miniplate fixation strategies.

**Fig. 10:**
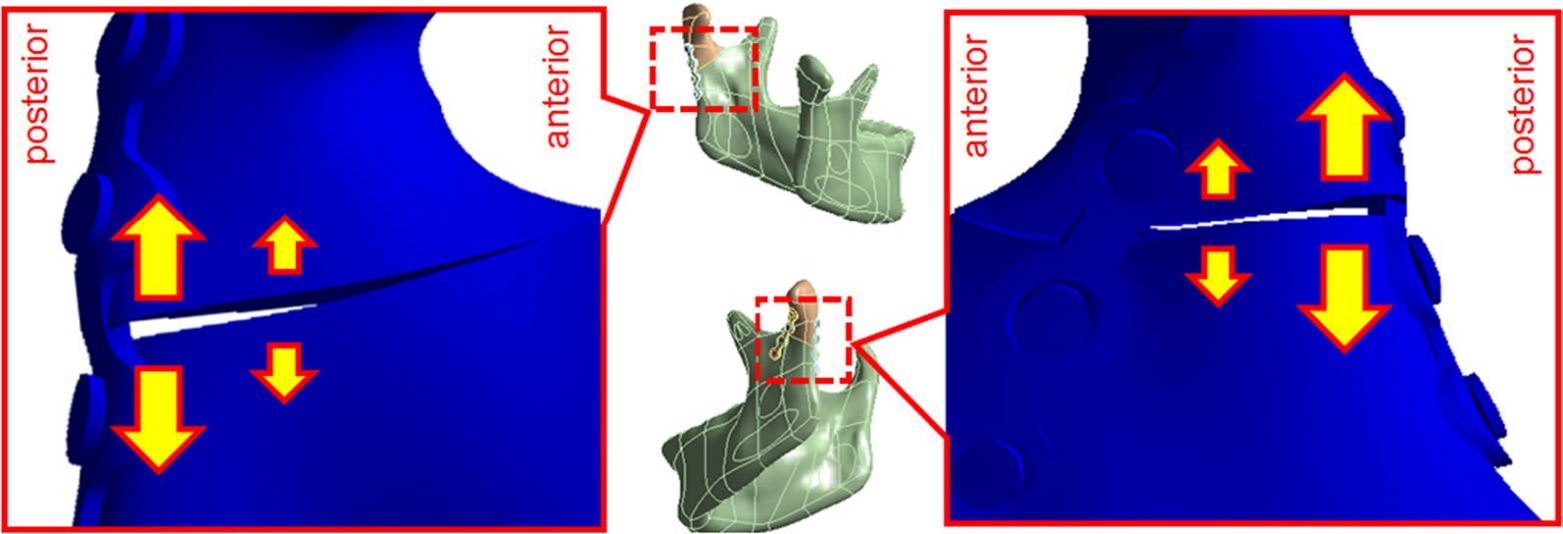
After occlusal force was applied, the interval between two segments was longer at posterior site than the anterior. (The figure showed the results of deformation magnified by 520 times)

When the stress distribution on the miniplates in each group was observed, high-stress areas were concentrated in the fracture line, in the lower half of the miniplate (Fig. 7). Additionally, when two miniplates were used for the fixation of the fracture area, the miniplate at the posterolateral surface (Fig. 1 position 2) or posterior surface of the subcondylar region (Fig. 1 position 3) had higher stress distribution compared to the miniplate at the anterolateral surface (Fig. 1 position 1) (Fig. 7). (23.07 MPa compared to 21.43 MPa in Group 4, 31.98 MPa compared to 22.38 Mpa in Group 5, 20.084 MPa compared to 13.28 Mpa in Gropu 6, 25.84Mpa compared to 13.46 Mpa in Group 7). It was consistent with Fig. 10, which showed a larger gap at the posterior side than the anterior side between two fracture fragments. The result implied that the fixed position of the posterior miniplate is subject to tension force and the site of anterior miniplate fixation (an auxiliary fixation site) is subject to compression force, the posterior part of the miniplate experiences higher stress.

Furthermore, higher bone stress disturbances on the fracture side were observed after reduced subcondylar fracture was fixed by miniplates (8.62–9.26 Mpa compared to 6.72 Mpa). (Fig. 8) The condition was consistent with the mandible functioning in the real world, which indicated that the mastication should be avoided due to the unhealing fracture might result in higher stress disturbance surrounding the fracture line and interfere with the healing process.

To evaluate the stability of different strategies of fixation, the sliding distance of the oblique surface of the mandibular condyle fracture site was observed. When the miniplate was implanted closer to the posterior portion of the subcondylar region, the sliding distance was smaller in the group where the miniplate was fixed closer to the posterior surface. (Group 3 (5.46 μm) than in Group 2 (6.24 μm); smaller in Group 5 (5.43 μm) than in Group 4 (6.10 μm); and smaller in Group 7 (5.76 μm) than in Group 6 (7.00 μm).) This was attributed to the posterior miniplate, which was fixed at the area where the tensile force was higher. Figure 10 showed how the two segments displaced after the occlusal force was applied. The interval was greater at the posterior side than the anterior side. It suggested that the force on the posterior side was tensile force. According to the previous study made by Champy [22], the ideal position of internal fixation was at the distribution of tensile force. Therefore, when miniplate was fixed closer to the posterior surface, the efficacy of fixation was better, and the sliding distance was more petite.

The structural stability was increased when two miniplates were used for fixation compared to only one. Therefore, the sliding distance of the oblique surface of the fracture site was slightly reduced. In this study, increasing the number of miniplates on the anterolateral surface of the subcondylar region (Fig. 1 position 1) reduced the sliding distance of the oblique surface of the fracture site in Group 4 (6.10 μm) to less than that of Group 2 (6.24 μm). Moreover, the sliding distance of Group 5 (5.43 μm) was less than that of Group 3 (5.46 μm). These results were consistent with the previous studies [9, 21, 23, 24], which showed less stability in using lesser miniplates for fixation. The results were also found in previous in vitro studies [25, 26] which stated that the stability was more excellent in subcondylar fracture fixed by two miniplates. Furthermore, using a longer miniplate for fixation reduces the sliding distance of the oblique surface of the fracture site in Group 4 (6.10 μm) to less than that of Group 6 (7.00 μm). Moreover, the sliding distance of Group 5 (5.43 μm) was less than that of Group 7 (5.76 μm).

Nevertheless, this study had several limitations. In the FEA, all materials were assumed to be homogeneous, isotropic, and linear elastic with their properties based on previous studies to simplify the simulation in this study and facilitate the comparison of results [27]. Thus, using these material property settings will not affect the general trend of the results, even if the results of the study are slightly different from the actual situation. Additionally, the computer model in this study included several simplifications, including that only the model was constructed, and the teeth were not included owing to differences in tooth structures and shapes. As the primary site of observation in this study was the TMJ, the teeth were not part of this primary site of observation; therefore, omitting them simplifies the evaluation. These simplifications were assumed not to have a severe impact on the results despite some differences from the actual situation. In addition, the model of this study was also validated with the cadaver study [28] and the result of the comparison showed similar trends.

This study used FEA to investigate the effects of subcondylar fracture fixation using miniplates at different positions and of different lengths. The results show that internal fixation of the miniplate on the posterior mandible resulted in a small amount of sliding motion on the oblique surface of the fracture site. Although the values analyzed in this study were slightly different from an actual clinical situation, the results provide a mechanical reference for clinicians and researchers. In the future, the results of this study can be used to conduct further mechanical studies related to miniplate placement, reduce the surgical failure rate, and achieve better outcomes for patients.

## Conclusions

In this study, a miniplate fixation strategy for subcondylar fracture suitable and convenient for open reduction was developed through FEA and using a bone plate that fulfills clinical needs. The results showed that miniplate fixation, which was placed closer to the posterior surface, could effectively reduce the amount of sliding in the fracture site, thereby achieving more excellent stability. Fixation efficiency was also improved with additional miniplate fixation at the anterior margin. The results aimed to provide clinicians with a biomechanical basis for positioning and miniplate length selection during miniplate implantation. If feasible (e.g., enough operating field, sufficient supporting bone for fixing additional miniplates), operators should always consider using two four-hole miniplates for internal fixation rather than one. The ideal position for the posterior miniplate was at the posterior surface of the mandible.

## Data Availability

All data generated or analysed during this study are included in this published article.
